# A Novel *mar*RAB Operon Contributes to the Rifampicin Resistance in *Mycobacterium smegmatis*

**DOI:** 10.1371/journal.pone.0106016

**Published:** 2014-08-25

**Authors:** Haiwei Zhang, Long Gao, Jiaoling Zhang, Weihui Li, Min Yang, Hua Zhang, Chunhui Gao, Zheng-Guo He

**Affiliations:** National Key Laboratory of Agricultural Microbiology, Center for Proteomics Research, College of Life Science and Technology, Huazhong Agricultural University, Wuhan, China; Indian Institute of Science, India

## Abstract

The multiple-antibiotic resistance regulator (MarR) plays an important role in modulating bacterial antibiotic resistance. However, the regulatory model of the *mar*RAB operon in mycobacteria remains to be characterized. Here we report that a MarR, encoded by Ms6508, and its *mar*RAB operon specifically contribute to rifampicin (RIF) resistance in *Mycobacterium smegmatis*. We show that the MarR recognizes a conserved 21-bp palindromic motif and negatively regulates the expression of two ABC transporters in the operon, encoded by Ms6509–6510. Unlike other known drug efflux pumps, overexpression of these two ABC transporters unexpectedly increased RIF sensitivity and deletion of these two genes increased mycobacterial resistance to the antibiotic. No change can be detected for the sensitivity of recombinant mycobacterial strains to three other anti-TB drugs. Furthermore, HPLC experiments suggested that Ms6509–Ms6510 could pump RIF into the mycobacterial cells. These findings indicated that the mycobacterial MarR functions as a repressor and constitutively inhibits the expression of the *mar*RAB operon, which specifically contributes to RIF resistance in *M. smegmatis*. Therefore, our data suggest a new regulatory mechanism of RIF resistance and also provide the new insight into the regulatory model of a *mar*RAB operon in mycobacteria.

## Introduction

Increase in bacterial drug resistance is a serious and major health concern worldwide. Recent studies have found that one important mechanism by which bacteria acquire multidrug resistance is the active efflux of drugs by multidrug transporters [1], [2]. To date, five multidrug transporter superfamilies have been reported in bacteria, of which ATP-binding cassette transporter (ABC) and major facilitator superfamily (MFS) are the two largest superfamilies [3]. Understanding the function and regulation of these multidrug transporters is vital in the fight against bacterial drug resistance worldwide.

A number of bacterial regulatory proteins, including repressors and activators, that govern the expression of drug transporters have been characterized [4]. The multiple-antibiotic resistance regulator (MarR) generally acts as a repressor of drug transporters [5], [6]. The MarR family is comprised of transcription factors that regulate genes involved in resistance to multiple antibiotics, organic solvents, oxidative stress agents and pathogenic factors [7]–[10]. The *Escherichia coli* MarR has been reported to bind to palindromic sequences within the *mar*O region between *mar*C and *mar*RAB [8], [11] and to negatively regulate the *mar*RAB operon [7]. Similarly, MexR has been found to negatively regulate an operon in *Pseudomonas aeruginosa* that encodes a multi-drug efflux system that results in increased resistance to multiple antibiotics [12], [13]. All MarR family members possess a common winged helix-turn-helix (HTH) motif responsible for their DNA-binding ability [14]), which can be altered by specific small molecules. For example, the *E. coli* MarR can be inactivated by salicylic acid, resulting in increased *mar*RAB expression and drug resistance [15]. OhrR, a homologue of MarR in *Xanthomonas ampestris,* derepresses transcription of the *ohr* gene when bound to hydrogen peroxide [16].

*Mycobacterium smegmatis* is a fast-growing mycobacterium that is used as a model strain for studying the gene regulatory mechanisms in mycobacterial species including pathogenic *M. tuberculosis,* the causative agent of tuberculosis (TB) [17], [18]. The *M. smegmatis* (GenBank accession number CP000480) genome encodes more than 500 potential regulatory factors, of which many are MarR regulators. In addition, the genomes of both *M. smegmatis* and *M. tuberculosis* encode at least two dozen putative drug efflux transporters [19] including various classes of bacterial drug exporters. The majority of these transporters belong to the two largest transporter families, MFS and ABC [4]. Some of these transporters have been shown to contribute to mycobacterial resistance to isoniazid (INH), rifampicin (RIF), tetracycline and other toxic compounds [1], [4], [20]. However, potential MarR regulators in mycobacteria and their effects on bacterial drug resistance remain to be clearly characterized.

In this study, we have characterized the function and regulatory mechanism of a novel MarR transcription factor, Ms6508, in *M. smegmatis*. Ms6508 acts as a repressor and negatively regulates the expression of two multidrug-like ABC transporter genes. Unexpectedly, we found that overexpression of two ABC transporters of the *mar*RAB operon significantly enhanced bacterial RIF sensitivity, whereas deleting them from the *M. smegmatis* genome resulted in RIF resistance. We have functionally characterized a *mar*RAB operon in which two ABC transporter genes specifically enhanced mycobacterial sensitivity to RIF. These observations are different from those of the known MarR-regulated multidrug transporters in other bacteria. Our findings provide the new insight into the regulatory model of *mar*RAB operon in mycobacteria.

## Results

### Ms6508 is a MarR-like regulator and Ms6508–6510 potentially encodes a *marRAB* operon

**Figure 1:**
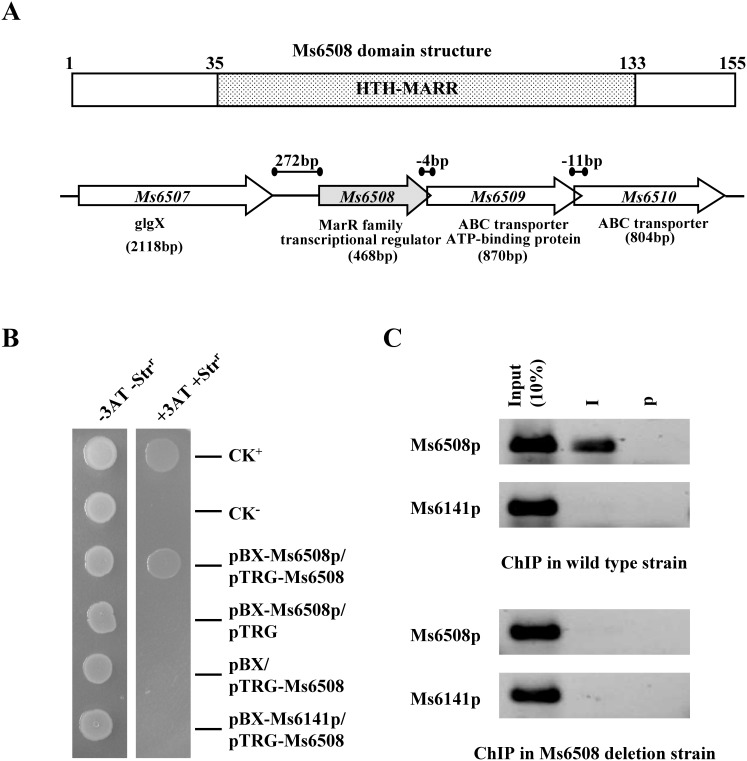
Analysis of the domain structure of MarR and its binding with its upstream DNA sequence. (**A**) Analysis of the domain structure of MarR and its genomic location. The *Ms6508* gene encodes a typical MarR regulator containing an HTH-MARR domain. Ms6508 shares a 272-bp common upstream promoter region with the Ms6509–6510 gene cluster. (**B**) Bacterial one-hybrid assays for the interaction between MarR and the upstream sequence of the *marRAB* operon. A pair of pBXcmT/pTRG plasmids was co-transformed into the reporter strain and its growth was monitored together with that of self-activation controls on selective medium. Co-transformants containing the pBX-Rv2031/pTRG-Rv3133 plasmids (24) served as positive controls (CK^+^) and co-transformants containing the empty vectors pBX and pTRG served as negative controls (CK^−^). Only the MarR+Ms6508p co-transformant strains and a positive strain CK^+^ grew well on the screening medium, indicating that MarR specifically interacts with the upstream sequence of the *marRAB* operon, Ms6508p. (**C**) ChIP assays in wildtype and *marR* deletion mutant *M. smegmatis* strains. ChIP using preimmune (P) or immune sera (I) raised against MarR. The mycobacterial promoter Ms6141p was used as a negative control.

**Figure 2:**
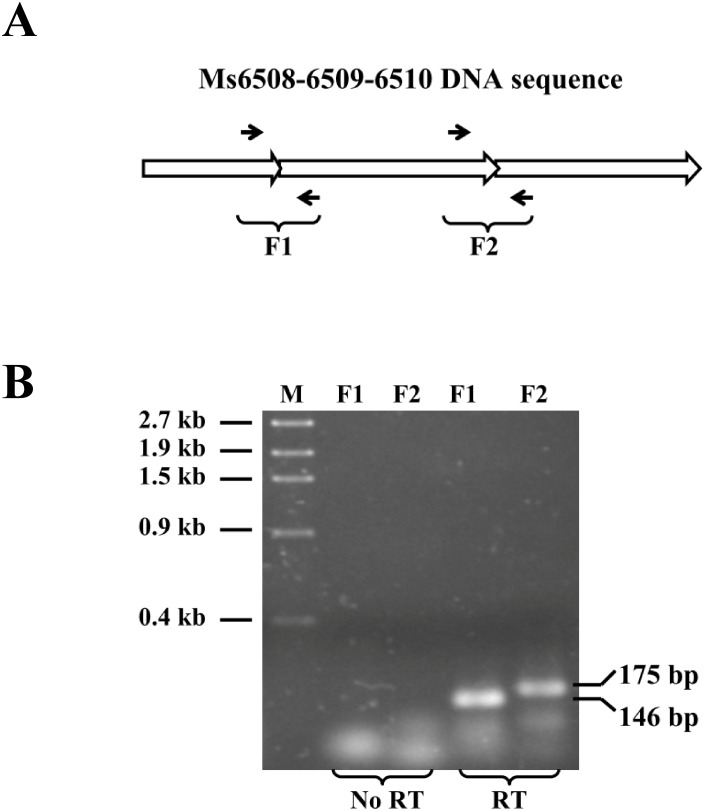
Assays for the Ms6508–Ms6510 co-transcription by reverse transcription PCR. (**A**) The operon structure of Ms6508–Ms6509–Ms6510. Primers were designed for assays and indicated by black arrows. (**B**) Reverse transcription PCR assays for Ms6508–Ms6510 co-transcription. mRNA (DNA-free) was used as negative controls. PCR procedure was as follows: reactions were degenerated at 95°C for 30 s, annealed at 60°C for 30 s, extended at 72°C for 30 s and under 35 cycles.

**Figure 3:**
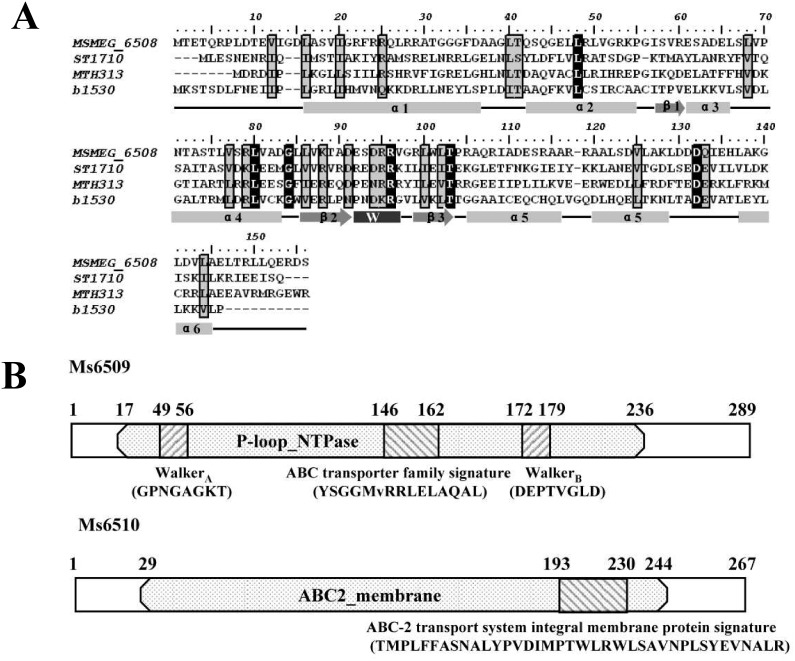
Sequence alignment and domain analysis of Ms6508–Ms6510. (**A**)The conserved amino acids residues of MarR are highlighted. ST1710, a MarR family regulator in *Sulfolobus tokodaii*; MTH313, a MarR family regulator in *Methanobacterium thermoautotrophicum*; b1530, a MarR family regulator in *E. coli.* (B) Domain assays for MarR and Ms6510. Ms6509 encodes a multidrug ABC transporter ATP-binding family protein and Ms6510 encodes a multidrug ABC transporter family protein.

*M. smegmatis* Ms6508 encodes a 155-residue protein containing a typical MarR helix-turn-helix (HTH) domain (Fig. 1A, upper panel). Genomic location analysis suggests that *marR* shares the same upstream DNA region with the *Ms6509–6510* operon (Fig. 1A). This idea could be confirmed by series of reverse transcriptional PCR assays and three genes were shown to co-transcript in *M. smegmatis* (Fig. 2). The *Ms6509–6510* operon is predicted to encode a multidrug ABC transporter ATP-binding protein (Ms6509) and a multidrug ABC transporter (Ms6510) (Fig. 3). This suggests that *Ms6508–6510* potentially encodes a *mar*RAB operon in *M. smegmatis.*

We examined the potential binding of MarR to the upstream region of the gene cluster. Using bacterial one-hybrid assays, the upstream DNA fragment was cloned into the reporter vector pBXcmT [21] and co-transformed with pTRG-Ms6508 into reporter strains. Only the Ms6508+Ms6508p co-transformant strains and a previously reported positive strain CK^+^ [21] grew well on the screening medium (Fig. 1B). No growth was observed for co-transformant strains containing Ms6508+Ms6141p, the negative control promoter, and for strains containing either Ms6508 alone or the upstream sequence alone.

We further carried out ChIP assays to investigate the specific binding of MarR to the upstream promoter of Ms6508–6510 *in vivo* in *M. smegmatis*. The target promoter could be specifically recovered by ChIP using an MarR antibody (Fig. 1C, upper panel). Importantly, a negative control promoter (Ms6141p) could not be recovered by MarR antisera. The specificity of MarR antibodies were confirmed using *marR* deletion strains (Fig. 1C, lower panel). These results established the upstream sequence of the operon as the target promoter of MarR in *M. smegmatis*.

### MarR binds with DNA fragments containing a palindromic sequence motif

**Figure 4:**
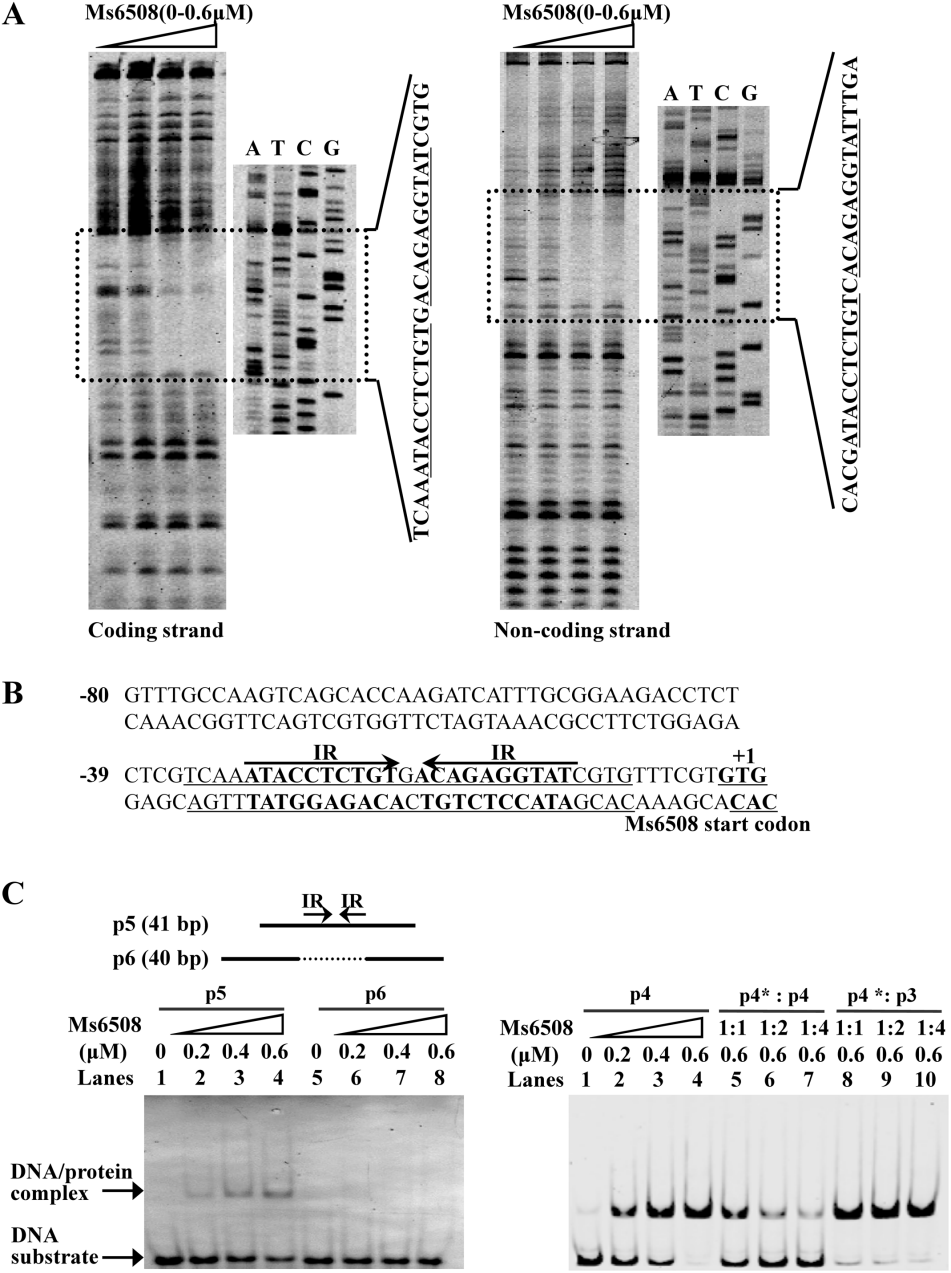
Identifying the DNA-binding motif of MarR. (**A**) DNaseI footprinting assays were carried out on the coding and non-coding strands. Protection of the promoter DNA by MarR against DNaseI digestion was tested by increasing the amount of MarR (0–0.6 µM). The ladders are shown and the corresponding nucleotide sequence is listed (lanes 2–4). The protected regions on the coding and non-coding strands are indicated. (**B**) Sequence and structural characteristics of the promoter DNA region protected by MarR. The regions protected by MarR are underlined. The 21-bp sequences containing the inverted repeats (IR) separated by 1 bp are indicated by a pair of arrows. The translation start codon of MarR is indicated in bold. (**C**) EMSA assays for the DNA-binding activity of MarR on DNA substrates with wildtype IR sequence and IR-deleted mutant sequences. DNA substrates were co-incubated with 0.2–0.6 µM of the MarR protein. Cold DNA substrate containing IR motif (p4), but not unrelated substrate (p3) which does not contain IR motif, could competitively inhibit the binding of MarR to the labeled DNA substrate (p4*).

We next characterized the binding motif recognized by MarR by DNase I footprinting assays. As shown in Figure 4A, when increasing amounts of MarR protein (0–0.6 µM) were co-incubated with DNase I, the region around TCAAATACCTCTGTGACAGA GGTATCGTG on the coding strand (Fig. 4A, right panel, lanes 2–4) and the region around CACGATACCTCTGTCACAGAGGTATTTGA on the noncoding strand (Fig. 4A, right panel, lanes 2–4) were clearly protected, indicating that these DNA regions contain a binding site for MarR. The protected DNA region extends from −35 to −7(relative to the translational start codon) in the coding strand (Fig. 4B) and contains a palindromic motif formed by two inverted repeats (IR, 5′-ATACCTCTGT-3′) separated from each other by one nucleotide (Fig. 4B).

EMSA assays on short synthesized oligonucleotides confirmed that the motif is required for specific recognition by MarR (Fig. 4C). We designed two DNA substrates in which one of the inverted repeats was deleted (Ms6508p-6) (Fig. 4C). As shown in Figure 4C (left panel), MarR was incapable of binding with the mutated substrate (Ms6508p-6). In contrast, MarR was able to bind the DNA substrate in which the conserved motif was not mutated. Further competition experiments also confirmed the specificity of MarR binding with the DNA substrate containing the inverted repeats. As shown in Figure 4C (right panel), the unlabeled DNA substrate containing the IR motif (lanes 5–7), but no unrelated substrate (lanes 8–10), could competitively inhibit the binding of MarR to the labeled DNA substrate. These results indicate that MarR recognizes and binds to an essential palindromic sequence motif.

We searched for the 21 bp sequence motif (ATACCTCTGTNACAGAGGTAT) in the rest of the *M. smegmatis* genome. However, no additional target gene was found, indicating that MarR specifically binds the upstream region of its operon.

### MarR is a repressor and negatively regulates expression of the *marRAB* operon genes

**Figure 5:**
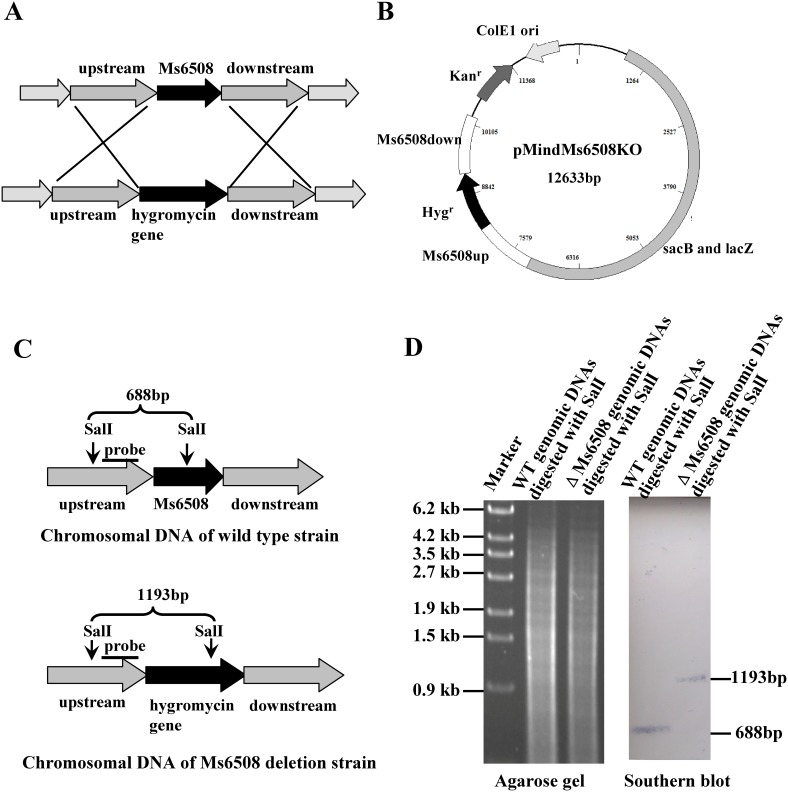
Construction of the MarR knockout strain of *M. smegmatis* and southern blot assays. (**A**) Schematic representation of the recombination strategy for removing *marR* from the genome of *M. smegmatis*. (**B**) A map of the recombinant vector pMindMs6508KO containing upstream and downstream sequences of *marR*, and the gene that confers resistance against hygromycin. (**C**) Schematic representation of the DNA fragments of the wildtype strain and the *marR* knockout strain treated with the restriction enzyme *SalI*. The probe is indicated with a black bar. (**D**) Southern blot assays. A 387 bp probe corresponding to the upstream sequences of *marR* in *M. smegmatis* was obtained by PCR and was labeled with digoxigenin dUTP (Boehringer Mannheim Inc., Germany).

We generated an *marR*-deletion mutant strain of *M. smegmatis* by a gene replacement strategy (Fig. 5) to further examine the regulatory effect of MarR. A knockout plasmid containing the Up and Down regions of the MarR gene and the selective hygromycin resistance gene (*Hgr*) was constructed and transformed into *M. smegmatis.* A Southern blot assay confirmed successful production of the marR-deletion mutant strain. As shown in Figure 5D, a signal band of about 1.2 kb was detected using a 387-bp probe from the *SalI*-digested genomic DNA of the mutant *M. smegmatis* strain. In contrast, a signal band of only about 0.7 kb was seen in the wildtype strain. This is consistent with the band sizes expected upon replacement of the *marR* gene with the Hygromycin^r^ gene and indicated that the *marR* gene was successfully deleted in the mutant strain.

**Figure 6:**
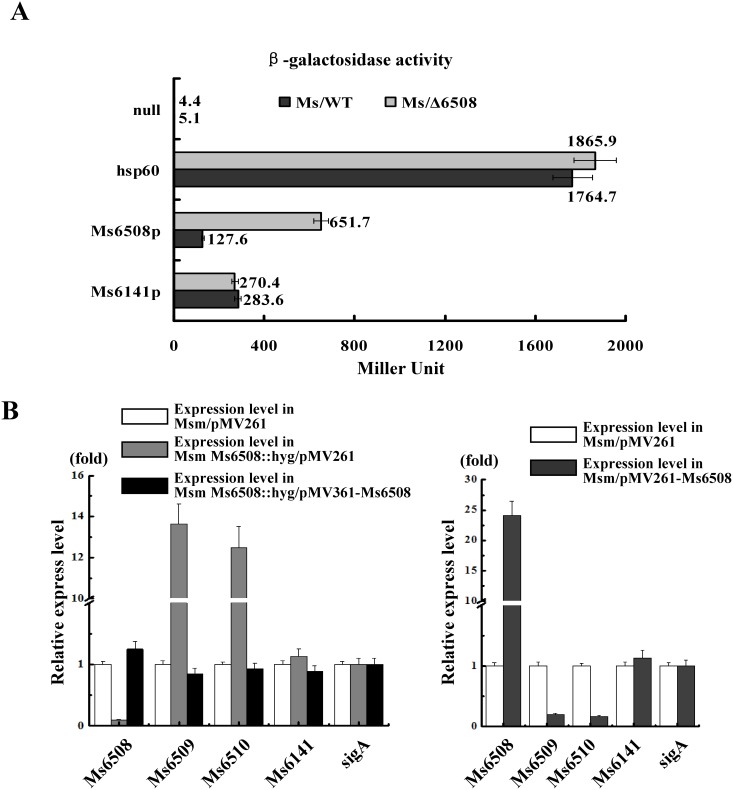
Expression assays for the effect of MarR on the target gene in wildtype and *marR* -deleted mutant strains. (**A**) Determination of β-galactosidase activity (Miller units). The values presented represent averages of three independent experiments. For statistical analysis, two-way ANOVA with Bonferroni multiple comparison tests were performed. P≤0.05 was considered statistically significant. (**B**) Quantitative real-time PCR assays for differential expression of the target genes in wildtype, mutant and complementation strains (left panel), and the *marR* overexpression strain (right panel). Relative expression levels of the genes were normalized using the *SigA* gene as an invariant transcript, and an unrelated Ms6141 gene as a negative control. Data were analyzed using the 2^ΔΔCt^ method [21]. Relative expression data were analyzed for statistical significance by the unpaired two-tailed Student’s T-test using GraphPad Prism (Version 5).

To further examine the regulatory effect of MarR on its target genes, a series of promoter-*lacZ* reporter plasmids was constructed using β-galactosidase as the reporter in *M. smegmatis.* As shown in Figure 6A, expression of *lacZ* was upregulated in the *marR*-deleted mutant *M. smegmatis* strain compared with the wildtype strain under the Ms6508p promoter. However, there was no significant difference in the expression of *lacZ* between the wildtype and mutant strains under the negative control promoter Ms6141p. These results indicated that MarR functions as a negative regulator in *M. smegmatis*.

A comparison of the expression levels of two target transporter genes in both wildtype and mutant mycobacterial strains was conducted using qRT-PCR assays. As shown in Figure 6B (left panel), compared with expression in the wildtype strain, two target genes (Ms6509 and Ms6510) were significantly upregulated (*P* value<0.01) in the *marR*-deleted mutant *M. smegmatis* strains. By contrast, expression of the negative control gene Ms6141 did not change significantly. Strikingly, when MarR was expressed in the mutant *M. smegmatis* strain, two target genes showed similar expression as that in the wildtype strain with empty pMV261 plasmid. This finding suggested that MarR can function as a repressor in *M. smegmatis*. Further overexpression assay confirmed this result. As shown in Figure 6B (right panel), expression of two genes was significantly downregulated (*P* value<0.01) when MarR was overexpressed using a pMV261-derived recombinant plasmid in *M. smegmatis* strains compared with the wildtype strain. This finding is consistent with the results of our β-galactosidase assays in the ΔMs6508 *M. smegmatis* strains described above.

Based on these results, we conclude that MarR functions as a repressor and negatively regulates expression of two target transporter genes (Ms6509 and Ms6510) in *M. smegmatis*.

### MarR positively regulates RIF resistance in *M. smegmatis*

**Figure 7:**
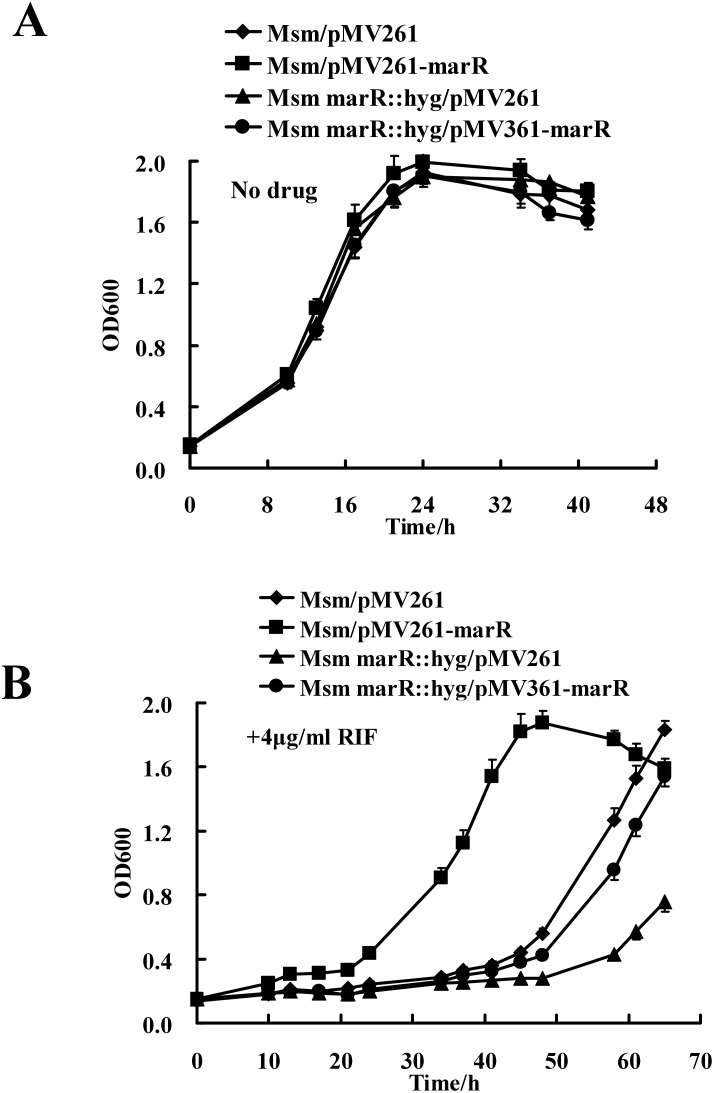
Assays for the effects of MarR on RIF resistance in *M. smegmatis*. Growth curves of the wild-ype, *marR*-overexpressed, deletion mutant and complementation strains were determined as described in Experimental Procedures. These mycobacterial strains were grown in 7H9 broth in the absence (**A**) and presence of 4 µg/ml RIF (**B**). Representative growth curves are shown.

*M. smegmatis* is naturally resistant to RIF [1], [22]. To examine the regulatory effect of MarR on RIF resistance in *M. smegmatis*, we further determined mycobacterial growth curves in response to the antibiotic. No obvious difference was observed in the growth of wildtype, *marR*-overexpressed, *marR*-deleted and complementation strains in 7H9 medium in the absence of RIF (Fig. 7A). However, compared with the wildtype strain, the *marR*-deleted *M. smegmatis* strain unexpectedly grew more slowly in 7H9 medium containing 4 µg/mL of RIF (Fig. 7B) and only a modest increase in the number of mycobacterial cells was observed. This result is in contrast to the known effects of other MarR regulators in *E. coli* [15]. Strikingly, when MarR was expressed using a pMV361 integration plasmid in the deletion *M. smegmatis* strain, the recombinant strain showed similar growth rate compared with the wildtype strain with the empty plasmid (Fig. 7B). Furthermore, the *marR*-overexpressed strain grew faster than the wildtype strain under the same conditions (Fig. 7B).

Thus, deletion of MarR decreased RIF resistance while overexpression of MarR increased RIF resistance in *M. smegmatis*. In contrast to negative regulation of target genes by other known bacterial MarR regulators, our results clearly showed that MarR positively regulates drug resistance in *M. smegmatis*.

### Two target genes, Ms6509 and Ms6510, negatively affect RIF resistance in *M. smegmatis*

Our data revealed that MarR repressed the expression of two target genes Ms6509 and Ms6510, but positively regulated mycobacterial RIF resistance. This implied that Ms6509 and Ms6510, two hypothetical ABC transporter genes, might play a negative role in drug resistance in *M. smegmatis*. To test this hypothesis, an Ms6509–6510 deletion mutant strain of *M. smegmatis* was first generated by a similar gene replacement strategy mentioned above.

**Figure 8:**
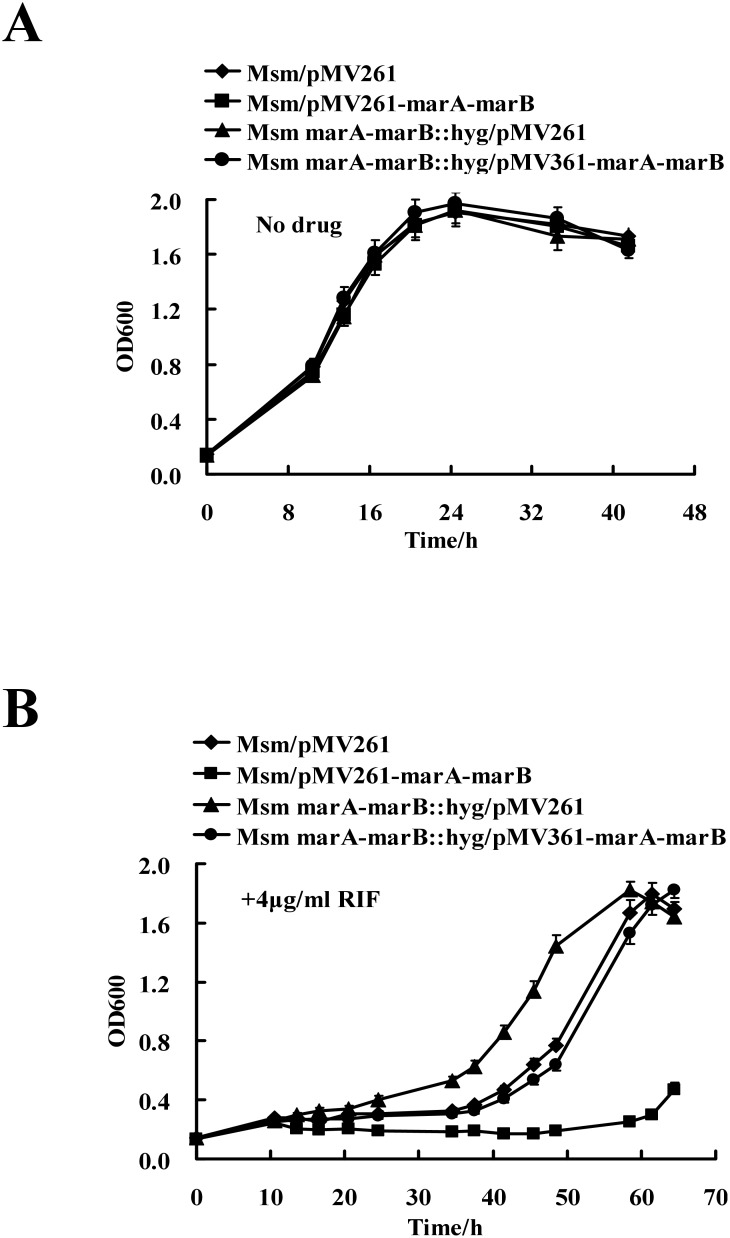
Effects of Ms6509–6510 genes on mycobacterial RIF resistance. (**A**) Growth curves of the wildtype, Ms6509–6510 overexpression, deletion mutant and complementation strains in 7H9 broth in the absence of RIF. (**B**) Growth curves of these mycobacterial strains in 7H9 broth in the presence of 4 µg/ml RIF.

We further determined mycobacterial growth curves and assayed the regulatory effect of Ms6509–6510 on the growth of *M. smegmatis* in response to RIF. No obvious difference was observed in the growth of wildtype, Ms6509–6510 overexpression, mutant, and complementation strains in the absence of RIF (Fig. 8A). However, the deletion mutant strain grew faster in 7H9 medium containing 4 µg/mL RIF (Fig. 8B), which was the same as the phenotype observed in *marR* overexpression strains (Fig. 7B). Strikingly, when the Ms6509–6510 genes were expressed in the mutant *M. smegmatis* strain, the recombinant strain showed similar growth rate as the wildtype strain with the empty pMV261 plasmid (Fig. 8B). In contrast, as in the case of *marR* deletion mutant strains (Fig. 7B), the Ms6509–6510 overexpression strains grew more slowly than the wildtype strain under the same conditions (Fig. 8B). We did not observe such effects on mycobacterial growth in the presence of two other anti-TB drugs, INH and streptomycin (data not shown). Thus, deletion of Ms6509–6510 specifically increased RIF resistance while overexpression of Ms6509–6510 decreased the resistance in *M. smegmatis*.

### Ms6509 and Ms6510 function as a drug transporter and can pump RIF into mycobacterial cells

**Figure 9:**
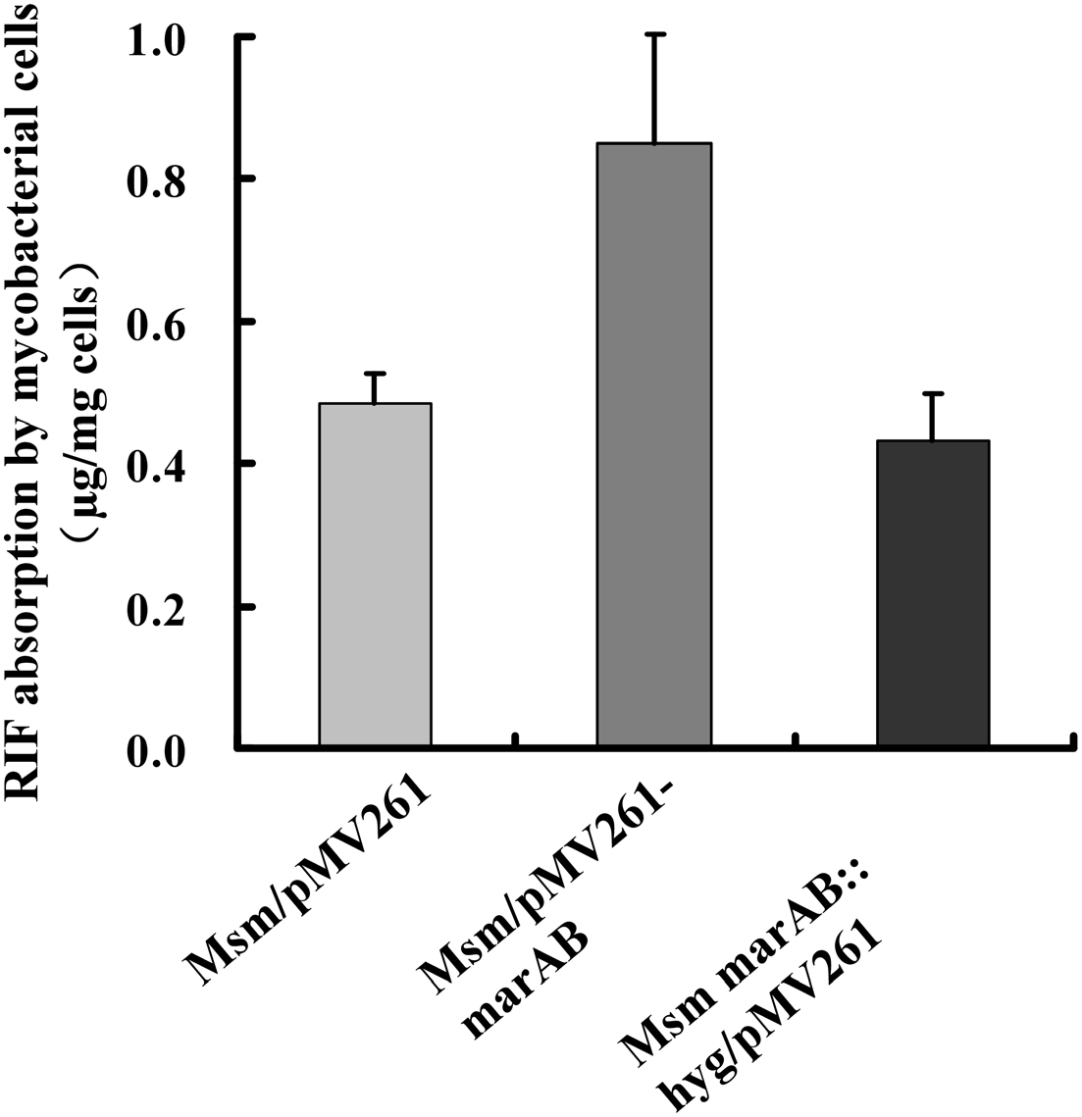
Determination of RIF transport and absorption by *M. smegmatis*. HPLC assays were performed as described in “Experimental Procedures”. The relationship of standard curve between RIF concentration and peak area was acquired through a series of concentration gradient standard RIF. A standard curve equation *Y* = 28403*X*−6721 (*r* = 0.9988) was obtained, in which *Y* represents peak area (µV×s), *X* represents RIF concentration (µg/ml) and *r* is the correlation coefficient. The samples peak area, from Msm/pMV261, Msm/pMV261-Ms6509–6510 and Msm Ms6509–6510: hyg/pMV261, was put into the standard curve equation and the RIF concentration of samples could be calculated. Each sample peak area was determined independently for three times. The quantity of the absorbed RIF by *M. smegmatis* was obtained by calculating the change of RIF concentration between two time points. Relative expression data were analyzed for statistical significance by the unpaired two-tailed Student’s T-test using GraphPad Prism (Version 5).

Above RIF resistance analysis on the Ms6509–Ms6510 overexpression and deletion strains suggested that two target genes could function as a RIF transporter in *M. smegmatis*. To test this idea, we used HPLC experiments to determine and compare the absorbed RIF amounts by several recombinant mycobacterial strains. As shown in Fig. 9, after 4-hr incubation at 37°C, about 0.85 mg RIF was transported and absorbed by each miligram cells of Ms6509–6510 overexpression mycobacterial strains. However, only about 0.45 mg RIF was transported by each miligram cells of wildtype (Msm/pMV261) or Ms6509–6510 deleted mutant strains under the same conditions. The *p* values of these absorbed RIF data were calculated and the difference between Ms6509–Ms6510 overexpression strains and wiltype or mutant strains was less than 0.05. Thus, it had statistical significance, indicating that Ms6509–6510 overexpression significantly enhanced mycobacterial RIF absorption. These results were consistent with our antibiotic resistance experiments above. Taken together, our data suggested that two target genes, Ms6509 and Ms6510, functioned as drug transporters and enhanced mycobacterial RIF sensitivity.

## Discussion

Several lines of evidence have shown that MarR is expressed widely in bacteria and regulates the expression of multidrug efflux pumps, which significantly contributes to bacterial drug resistance [1]–[3]. Although the crystal structure of a MarR regulator has been recently reported [22] in *M. tuberculosis*, the regulatory model of a *mar*RAB operon remains to be clearly characterized in mycobacteria. In the present study, we have identified a *mar*RAB operon, in which a MarR regulator, encoded by Ms6508, negatively regulates the expression of two ABC transporters, encoded by Ms6509–6510. Importantly, we provide evidence that the MarR-regulated transporter in *M. smegmatis* has a novel and unique function compared to other known *mar*RAB operons.

The *M. smegmatis* mc^2^155 genome encodes more than 500 transcriptional factors, of which 22 are potential MarR regulators and two dozen are putative drug exporters (GenBank accession number CP000480). However, only one MarR, encoded by MarR, has been found in the *mar*RAB operon (Ms6508–Ms6510). In the current study, using gene knockout and β-galactosidase activity assay as well as qRT-PCR experiments, we found that MarR functions as a MarR repressor and negatively regulates the expression of Ms6509 and Ms6510 in the operon. Similar to previously reported *Escherichia coli* MarR [8], [11], [15], the *M. smegmatis* MarR was also found to recognize a palindromic sequence within the upstream promoter region of *mar*RAB. Our data suggested that the newly identified MarR regulator represented a typical MarR transcriptional factor in *M. smegmatis*. However, we unexpectedly found that the MarR-regulated ABC transporters in the unique *mar*RAB operon did not function as multidrug efflux pumps as reported so far in other bacteria. In the case of other known multidrug transporters regulated by a MarR regulator, drug resistance increases with the expression level of the transporters [8], [11], [15]. However, we found that overexpression of Ms6509 and Ms6510 remarkably decreased mycobacterial RIF resistance and deletion of these two transporter genes resulted in increased RIF resistance in *M. smegmatis*. These results led us to characterize Ms6508–Ms6510 as a unique MarR-regulated *mar*RAB gene cluster in *M. smegmatis*.

It has been reported that *M. smegmatis* is naturally resistant to RIF even though no mutations have been characterized in the RIF resistance-determining region of the *rpoB* gene [1], [23]. Although some evidence has shown that RIF inactivation via ribosylation may be a principal mechanism of RIF resistance in *M. smegmatis*, this mechanism cannot solely account for its high level of RIF resistance [1], [23]. It has been demonstrated that porin MspA plays an important role in the influx of quaternary ammonium compounds and antibiotics in *M. smegmatis* [24]; however, the efficacy of rifampicin against *M. smegmatis* is not strongly affected by the absence of porin [25], implying that other unknown mechanisms are involved in the regulation of rifampicin resistance in *M. smegmatis*. In the present study, we report that a MarR and its regulation of the *mar*RAB operon contribute to RIF resistance in *M. smegmatis*. Unlike other known drug efflux pumps, expression of these two ABC transporters increased RIF sensitivity. This indicated to us that MarR functions as a repressor and constitutively inhibits their expression, which contributes to RIF resistance in *M. smegmatis*. Interestingly, *mar*RAB appears to be specifically involved in regulation of RIF resistance, as we did not observe any change in the sensitivity of recombinant mycobacterial strains to three other anti-TB drugs. Thus, our data reveal a new regulatory mechanism of RIF resistance in *M. smegmatis*.

In summary, we have functionally characterized a novel MarR regulator in mycobacteria and its unique physiological function. We provide evidence to show that MarR is involved in the regulation of two ABC transporter genes and specifically enhances rifampicin resistance in *M. smegmatis*. MarR represents a typical MarR transcriptional factor in *M. smegmatis*. However, we show that the MarR-rgulated ABC transporters in the *mar*RAB operon in *M. smegmatis* are functionally different from the multidrug transporters of known *mar*RAB operons in other bacteria. Our findings provide the new insight into the function of both MarR-regulated transporters and the *mar*RAB operon in mycobacteria.

## Materials and Methods

### Strains, Enzymes, Plasmids and Chemicals

*Escherichia coli* BL21 strains and the pET28a expression vector were purchased from Novagen. The pBT and pTRG vectors, *E. coli* XR reporter strains, and reagents for the one-hybrid assay were obtained from Stratagene. All enzymes including DNA polymerase, restriction enzymes and DNA ligase, deoxynucleoside triphosphates (dNTPs), and all antibiotics were purchased from TaKaRa Biotech. PCR primers were synthesized by Invitrogen. All plasmids constructed for the purposes of our study are listed in Table S1.

### Cloning, expression and purification of recombinant proteins

*M. smegmatis* mc^2^155 genes were amplified by PCR from mycobacterial genomic DNA using their respective primers. The *marR* gene was amplified and cloned into the expression vector pET28a to produce recombinant proteins. Recombinant *E. coli* BL21 cells were grown in 1L LB medium up to OD_600_ of 0.6 at 37°C. Protein expression was induced by the addition of 1 mM isopropyl β-D-1- thiogalactopyranoside (IPTG) at 37°C for 10 h. Proteins were purified on Ni^2+^ affinity columns as described previously [26] and the elute was dialyzed overnight and stored at –80°C.

### Bacterial one-hybrid and electrophoretic mobility shift assays (EMSA)

The bacterial one-hybrid assay was carried out as described previously [21]. The regulatory sequences of the *marR* and Ms6141 gene, Ms6508p and Ms6141p, respectively, were cloned into the reporter vector pBXcmT [21]. *marR* was fused into the N-terminal domain of the α-subunit of RNA polymerase in the pTRG vector (Stratagene).

The upstream fragments of the *marR* and Ms6141 genes were amplified by PCR from *M. smegmatis* mc^2^155 genomic DNA or short oligonucleotides were synthesized for DNA-binding activity assays. DNA fragments used in this assay were labeled with fluorescein isothiocyanate (FITC). The reactions (10 µl) for measuring mobility shift contained labeled DNA substrates incubated with increasing concentrations of the protein (as indicated in the legend of the corresponding figure) in EMSA buffer (100 mM Tris-HCl, pH 8.0, 100 mM NaCl, 1 mM DTT and 10% glycerol) for 30 min at room temperature before analyzing by 5% native polyacrylamide gel electrophoresis (PAGE). Electrophoresis and imaging of gels were performed as described previously [26].

### DNase I footprinting Assays

DNaseI footprinting was performed as described previously [27]. The 148 bp upstream promoter regions of the *marR* gene (coding and non-coding strands) were amplified by PCR using primers labeled with FITC. The products were purified and subjected to the same binding reaction as in EMSA. After that, DNase I and its 10×reaction buffer (Takara) were added to the reaction mixture. Incubation was continued for about 3 min, and an equal volume of stop buffer (1% sodiumdodecyl sulfate, 200 mM NaCl, and 20 mM EDTA) was added. The reaction mixture was extracted with phenol-chloroform, ethanol precipitated, then resuspended in 10 µl H_2_O. DNA ladders were produced using the Sanger dideoxy method. All samples were analyzed by PAGE on 8% denatured gel with 8 M urea. Images were acquired using the Typhoon scanner (GE Healthcare).

### Chromatin immunoprecipitation (ChIP) analysis

*In vivo* interactions between MarR and its target promoters in *M. smegmatis* were investigated by ChIP assays. *M. smegmatis* cells were fixed with 1% formaldehyde and cross-linked cells were resuspended in 1 mL of TBSTT (TBS 0.2% Triton X-100 0.05% Tween-20). The bacterial sample was sonicated on ice and then incubated with preimmune or immune sera raised against the MarR protein under rotation for 3 h at 4°C. The complexes were immunoprecipitated with 20 µL 50% proteinA-agarose for 1 h. The immunocomplex was recovered and cross-linking was reversed for 6 h at 65°C. DNA recovered from immunoprecipitates was amplified with primers specific for the *marR* promoter or the Ms6141 promoter, which was used as a negative control.

### Construction of the gene deletion mutant of *M. smegmatis* and Southern blot analysis

Knockouts of the *marR* and Ms6509–6510 genes from *M. smegmatis* mc^2^155 were performed as described previously [27]. A pMind-derived [28] suicide plasmid, pMindMs6508KO for *marR* knockout or pMindMs6509–6510KO for Ms6509–6510 knockout, carrying a hygromycin resistance gene was constructed and a *sacB*-*lacZ* gene was inserted to confer sensitivity to sucrose as a negative selection marker. The gene knockout strain was selected on LB agar medium containing 50 µg/mL hygromycin, 2% sucrose and 200 µg/mL X-Gal. Deletion of the *marR* or Ms6509–6510 genes was confirmed by Southern blot analysis as described previously [27].

### Construction of *marR* or Ms6509–6510 overexpression strains and complementation strains

*M. smegmatis marR* and Ms6509–6510 genes were amplified by PCR from mycobacterial genomic DNA using their respective primers. *marR* and Ms6509–6510 were then inserted downstream of the strong *hsp60* promoter of pMV261 [29] for overproducing these genes in *M. smegmatis*. The *marR* or Ms6509–6510-complemented *M. smegmatis* strains were constructed by integrating *marR* or Ms6509–6510 gene into the chromosomes of the respective deletion strains. The *marR* or Ms6509–6510 gene was first cloned into a pMV361 vector [28], and the recombinant plasmid pMV361-Ms6508 or pMV361-Ms6509–6510 was transformed into the respective *M. smegmatis* mutant strains. The complementation strain was selected on 7H10 medium (complemented with 0.2% glycerol) containing 30 µg/mL kanamycin.

### Analysis of β-galactosidase activity

β-galactosidase activity assays were performed in *M. smegmatis* by creating operon-*lacZ* fusions based on the expression vector pMV261 [27]. The upstream sequences of *marR* or Ms6141 were first cloned into the pMV261 backbone, then the reporter gene *lacZ* was cloned immediately downstream. Null promoter-*lacZ*, Ms6141p-*lacZ*, and hsp60-*lacZ* were used as controls. The reporter plasmids were transformed into wild type strains and the mutant ΔMs6508 strain to obtain the corresponding recombinant reporter strains. All strains were grown in 7H9-Tw-glycerol-Kan medium at 37°C for 48 h [17]. Some cell suspensions were then inoculated into 7H9-Tw-glycerol-Kan liquid medium and grown at 37°C to OD_600_ of 0.5 to 0.8. β-Galactosidase measurements were performed as described previously [27]. Others cell suspensions were plated on 7H10-glycerol-Kan-X-gal solid medium and grown at 37°C for imaging.

### Quantitative real-time PCR assays

Isolation of mRNA and cDNA preparation of wildtype strains, overexpression strains, deletion mutants and complementation *M. smegmatis* strains were performed and real-time PCR analysis was subsequently carried out according to previously described procedures [26]. The reactions were performed in a Bio-Rad IQ5 RT-PCR machine under the following thermocycling conditions: 95°C for 5 min and 40 cycles at 95°C for 30 s, 60°C for 30 s and 72°C for 30 s. Amplification specificity was assessed using melting curve analysis. Gene expression levels were normalized to the levels of *sigA* gene transcripts. An unrelated Ms6141 gene was used as a negative control. The degrees of expression change were calculated using the 2^−ΔΔCt^ method [26]. Average relative expression levels and standard deviations were determined from three independent experiments.

### Determination of mycobacterial growth curves and the effect of antibiotics

Growth patterns of the wildtype mycobacterial strain, the gene deletion mutant, the overexpressing strain, and complementation strains were examined according to the procedures described previously [17] with some modifications. To determine mycobacterial growth curves and the effect of antibiotics, *M. smegmatis* was grown overnight in Middlebrook 7H9 broth (supplemented with 0.05% Tween80 and 0.2% glycerol) containing 50 µg/mL Hyg and 30 µg/mL Kan. When cells entered a stationary growth phase (OD_600_ between 1.5 and 2.0), each culture was diluted (4∶100) in 100 mL of fresh 7H9 broth containing the indicated concentration of each antibiotic. Hyg was not added in the 7H9 medium when assaying RIF resistance of overexpression or gene deletion mutant strains. The cultures were then allowed to grow further at 37°C with shaking at 160 rpm. Aliquots were taken at the indicated times for determining OD_600_.

### Determination of RIF transport and absorption by *M. smegmatis*

RIF transport and absorption by mycobacterial strains were determined by HPLC [30]. *M. smegmatis* was grown to an OD_600_ of 1.2 in 7H9 media. RIF was added to the cultures to a final concentration of 4 µg/ml. 3 ml cultures were removed as a negative control. The rest of cultures were incubated for additional 4 hours at 37°C. Another 3 ml cultures was removed and the supernatants were collected by centrifuge at 12000 rpm for 3 min. Then, these supernatants from two different time points were filtered through a 0.22-µm filter, respectively, and 10 µl was injected into C-18 column for HPLC analysis and separated by reverse-phase HPLC (Shimadzu LC-20AT). The mobile phase consists of methanol–monopotassium phosphate (pH 5.8; 10 mM) (65∶35, v/v). Chromatography was performed at 25°C at a flow rate of 0.6 ml/min with a UV detector at 254 nm wavelength. The samples peak area for Msm/pMV261, Msm/pMV261-Ms6509–Ms6510 and Msm Ms6509–6510:: hyg/pMV261 (0 h and 4 h in RIF) was determined independently for three times, respectively. The amount of the transported and absorbed RIF by *M. smegmatis* was obtained by calculating the difference of RIF concentration in the supernatants between two different time points.

## Supporting Information

Table S1 **Strains and plasmids used in this study.**
(DOC)
