# Supplementary material for: A Novel marRAB Operon Contributes to the Rifampicin Resistance in Mycobacterium smegmatis
Source: PLoS One. 2014 Aug 25;9(8):e106016. doi: 10.1371/journal.pone.0106016 (PMC4143341; doi:10.1371/journal.pone.0106016)
Supplement: Table S1 — Strains and plasmids used in this study. (DOC) [file pone.0106016.s001.doc]

**Table S1 Strains and plasmids used in this study**

| Plasmid or Strain | Relevant genotype or features | Source or reference |
| --- | --- | --- |
| *E. coli* |  |  |
| DH5a | Host for plasmid construction | TaKaRa |
| BL21 | Host for overexpression | TaKaRa |
| XR | Host for bacteria one-hybrid | Stratagene |
| *M. smegmatis mc^2^155* |  |  |
| Msm/WT | *M. smegmatis* |  |
| Msm/pMV261 | mc^2^155with pMV261 | This study |
| Msm/pMV261-Ms6508 | mc^2^155with pMV261:: Ms6508 | This study |
| Msm/Ms6508::hyg | mc^2^155 Ms6508 replaced by hyg | This study |
| Msm Ms6508::hyg /pMV361-Ms6508 | Msm Ms6508::hyg with pMV361:: Ms6508 | This study |
| Msm/pMV261-Ms6509-6510 | mc^2^155with pMV261:: Ms6509-6510 | This study |
| Msm/Ms6509-6510::hyg | mc^2^155 Ms6509-6510 replaced by hyg | This study |
| MsmMs6508::hyg/pMV361-Ms6509-6510 | Msm Ms6508::hyg with pMV361:: Ms6509-6510 | This study |
| Msm Ms6508::hyg/pMV261-LacZ | Msm/Ms6508::hyg with pMV261::LacZ | This study |
| Msm Ms6508::hyg/pMV261-hsp60-LacZ | Msm/Ms6564::hyg with pMV261::hsp60::LacZ | This study |
| Msm Ms6508::hyg/pMV261-6508p-LacZ | Msm/Ms6508::hyg with pMV261::6508p::LacZ | This study |
| Msm Ms6508::hyg/pMV261-6141p-LacZ | Msm/Ms6508::hyg with pMV261::6141p::LacZ | This study |
| Msm/pMV261-LacZ | mc^2^155 with pMV261::LacZ | This study |
| Msm/pMV261-hsp60-LacZ | mc^2^155 with pMV261::hsp60::LacZ | This study |
| Msm/pMV261-Ms6508p-LacZ | mc^2^155 with pMV261:: Ms6508p::LacZ | This study |
| Msm/pMV261-Ms6141p-LacZ | mc^2^155 with pMV261:: Ms6141p::LacZ | This study |
| pET28a(+) | Kan^r^, T7 lac promoter, N-terminal His6 | Novagen |
| pET28a-Ms6508 | Ms608 in EcoRI-XbaI sites of pET28a | This study |
| pBXcmT | chlo^r^, p15A replicon, lac-UV5 promoter | 20 |
| pBX-Ms6508p | Ms6508p in EcoRI-XbaI sites of pBXcmT | This study |
| pBX-Ms6141p | Ms6141p in EcoRI-XbaI sites of pBXcmT | This study |
| pTRG | tet^r^, ColE1 replicon, lpp/lac-UV5 promoter | Stratagene |
| pTRG-Ms6508 | Ms6508 in EcoRI-XbaI sites of pTRG | This study |
| pMV261 | Kan^r^, pAL5000 replicon |  |
| pMV361 | Kan^r^, no pAL5000 replicon |  |
| pMV261-Ms6508 | Ms6508 in EcoRI-XbaI sites of pMV261 | This study |
| pMV261-Ms6509-6510 | Ms6509-6510 in EcoRI-XbaI sites of pMV261 | This study |
| pMV361-Ms6508 | Ms6508 in EcoRI-HindⅢ sites of pMV361 | This study |
| pMV361-Ms6509-6510 | Ms6509-6510 in EcoRI- HindⅢ sites of pMV261 | This study |
| pMV261-LacZ | LacZ in HindⅢ-NheI sites of pMV261 | This study |
| pMV261-hsp60-LacZ | hsp60 in EcoRI-HindⅢsites of pMV261:: LacZ | This study |
| pMV261-Ms6508p-LacZ | Ms6508p in EcoRI-HindⅢsites of pMV261:: LacZ | This study |
| pMV261-Ms6141p-LacZ | Ms6141p in EcoRI-HindⅢsites of pMV261:: LacZ | This study |
